# Anti-Ageing Effect of *Physalis alkekengi* Ethyl Acetate Layer on a d-galactose-Induced Mouse Model through the Reduction of Cellular Senescence and Oxidative Stress

**DOI:** 10.3390/ijms21051836

**Published:** 2020-03-06

**Authors:** Kaiyue Sun, Yingting Sun, Heyang Li, Dongyao Han, Yuting Bai, Rong Zhao, Zijiao Guo

**Affiliations:** College of Animal Science and Veterinary Medicine, Shanxi Agricultural University, Taigu 030801, Shanxi, China; syt09809@163.com (Y.S.); liheyangnongda@163.com (H.L.); handongyaoyao@163.com (D.H.);

**Keywords:** *Physalis alkekengi*, learning and memory, exercise endurance, cellular senescence, oxidative stress

## Abstract

We aimed to study the effects of an ethyl acetate fraction of *Physalis alkekengi* (PAE) on d-galactose (d-gal)-induced senescence and the underlying mechanism. Firstly, analysis of the phytochemical composition revealed total flavonoids, total phenolics, total saponins, rutin, and luteolin contents of 71.72 ± 2.99 mg rutin equivalents/g, 40.19 ± 0.47 mg gallic acid equivalents/g, 128.13 ± 1.04 mg oleanolic acid equivalents/g, 1.67 ± 0.07 mg/g and 1.61 ± 0.01 mg/g, respectively. The mice were treated with d-gal for six weeks, and from the fifth week, the mice were administered with PAE by gavage once a day for five weeks. We found significant d-gal-induced ageing-related changes, such as learning and memory impairment in novel object recognition and Y-maze, fatigue in weight-loaded forced swimming, reduced thymus coefficient, and histopathological injury of the liver, spleen, and hippocampus. The PAE effectively protected from such changes. Further evaluation showed that PAE decreased the senescence-associated *β*-galactosidase of the liver, spleen, and hippocampus, as well as the oxidative stress of the liver, plasma, and brain. The abundance of flavonoids, phenols, and saponins in PAE may have contributed to the above results. Overall, this study showed the potential application of PAE for the prevention or treatment of ageing-associated disorders.

## 1. Introduction

Ageing-related diseases are attracting widespread attention as the global population increases and human lifespan extends. By 2050, the proportion of the world’s population over 60 years old will reach 22% [1]. Ageing is an irreversible process characterized by various progressive bouts of degeneration of physiological functions. The morbidity and death rate of humans increase with ageing. Moreover, risk factors for humans increased with ageing to develop various chronic diseases, such as Alzheimer’s disease (AD) [2], fatigue [3], tumor suppression [4], type 2 diabetes, obesity [5], and cardiovascular disorders [6].

DNA damage and cellular senescence caused by free radicals are two primary mechanisms for age-related disease [7]. Furthermore, several studies have shown that reduction of cellular senescence could improve the healthspan [8] and ameliorate age-related diseases, such as hepatic steatosis [9], post-traumatic osteoarthritis [10], Parkinson’s disease [11], and AD [12]. The Food and Drug Administration have proved six substances for the anti-ageing clinical study; five are related to the reduction of senescent cells or senescence-associated secretory phenotype [13]. These conditions suggest that senescent cells are an emerging target for ageing-related diseases, and inhibition of senescent cells could delay ageing. Some active compounds, such as metformin, rapamycin, or senolytics that targeted to senescent cells, are found to slow down the ageing-relative diseases, such as cardiac dysfunction, loss of muscle mass, and osteoporosis [14,15,16]. However, their value is limited because of disadvantages, such as hyperlipidemia and fatigue [17,18]. Thus, researchers are actively developing novel substances with good efficacies and few side effects [15].

*Physalis alkekengi* L. var. *Franchetii*, named the Chinese lantern, is an edible plant classified in the Solanaceae family. The calyx and fruit of *P. alkekengi* L. var. *franchetii* (Mast.) *Makino* possess a variety of properties, including anti-inflammatory, antimicrobial, antitumor, and anti-diabetes [19,20,21,22,23]. The ethyl acetate fraction of *P. alkekengi* (PAE) inhibited lipopolysaccharide-induced pro-inflammatory mediators in BV2 cells [24] and demonstrated potent memory improvement in scopolamine-induced cognitive impairments through anti-oxidative stress [25]. However, PAE’s effect on ageing has not been explored. In this study, we used a d-galactose (d-gal)-induced senescence mouse model to observe the protective effect of the PAE.

## 2. Results

### 2.1. Component Analysis of PAE

**Figure 1:**
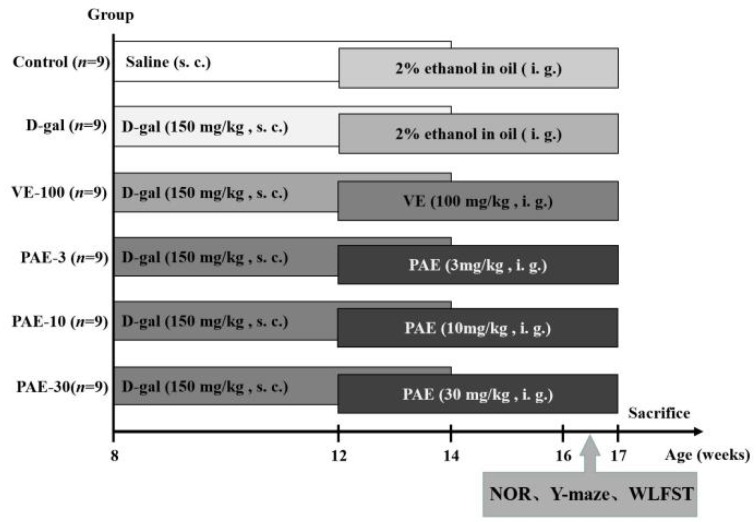
Experimental design and timeline.The mice were treated with once-daily d-galactose (d-gal) (150 mg/kg) or saline by subcutaneous injection for the first six weeks. From the fifth week, the vitamin E (VE) or *Physalis alkekengi* (PAE) groups were subjected to VE (100 mg/kg) or PAE (at doses of 3, 10, 30 mg/kg, oral) once daily for five weeks, and the control group and model group were administered a solvent (2% ethanol in oil, oral) in the same way instead. In the ninth week, the behavior tests were investigated after PAE administration. NOR, novel object recognition; WLFST, weight-loaded swimming test.

**Figure 2:**
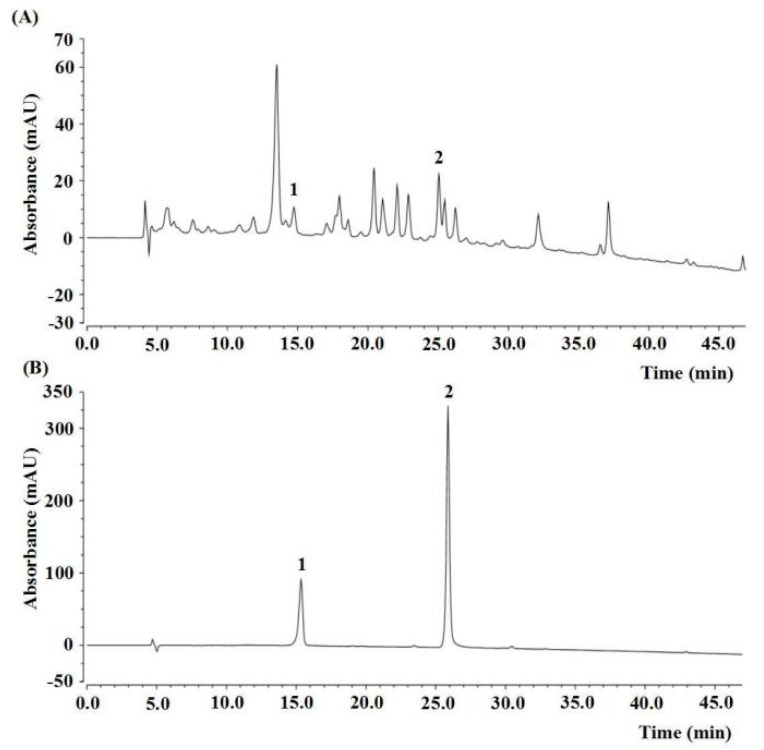
HPLC chromatogram of the ethyl acetate fraction of *Physalis alkekengi*. (**A**) HPLC analysis of PAE, and (**B**) external standard. The peak 1, rutin (t’_R_ = 10.32 min); peak 2, luteolin (t’_R_ = 20.84 min).

The experimental design and timeline are shown in Figure 1. The contents of active components in PAE are shown in Table 1. The total flavonoid content (TFC), total phenolic content (TPC), and total saponins content (TSC) were 71.72 ± 2.99 mg rutin equivalents (REs)/g, 40.19 ± 0.47 mg gallic acid equivalents (GAEs)/g and 128.13 ± 1.04 mg oleanolic acid equivalents (OAEs)/g, respectively. The contents of rutin and luteolin in PAE were 1.67 ± 0.07 mg/g and 1.61 ± 0.01 mg/g, respectively, calculated by high-performance liquid chromatography (HPLC) and by using an external standard (Table 1, Figure 2).

### 2.2. PAE Improved the d-gal-Induced Recognition Decline

**Figure 3:**
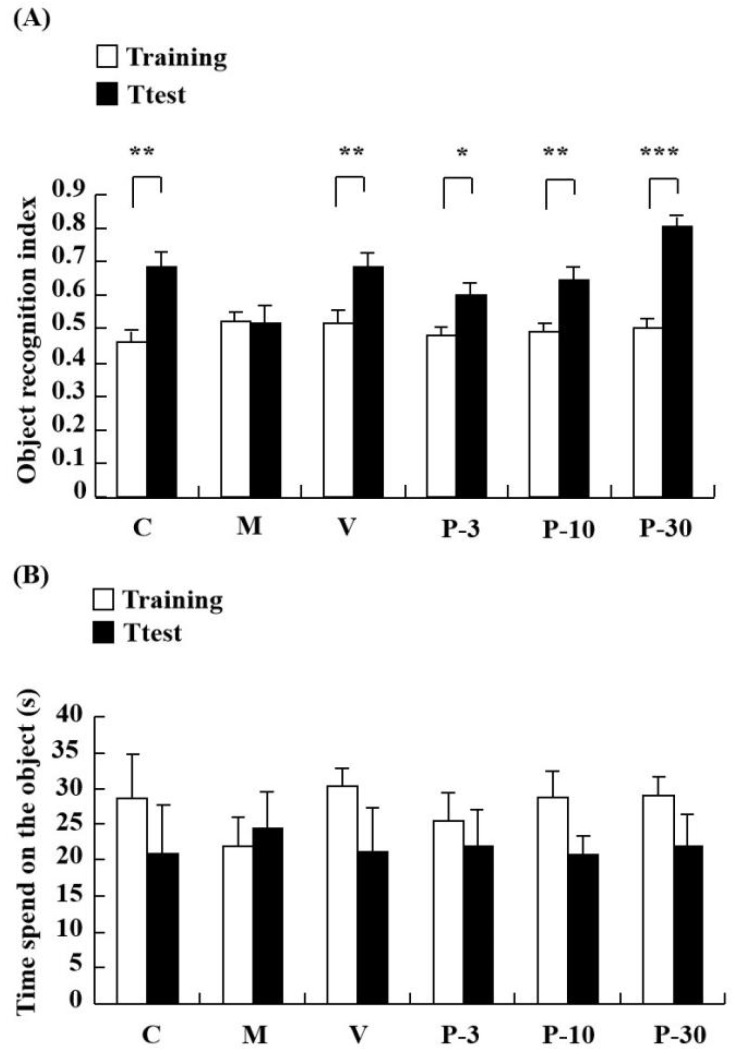
PAE effects on d-gal-induced memory impairment in the novel object recognition (NOR) test. The mice were placed into the apparatus containing two identical objects and allowed to explore for 5 min in the training session. After 60 min, a novel object replaced one of two identical objects, and then the test session was performed. The object exploring time of each mouse in the training session and test session were recorded. (**A**) Object recognition index and (**B**) time spent on the object in seconds in NOR. Results are presented as mean ± SEM. *n* = 9, * *p* < 0.05, ** *p* < 0.01, *** *p* < 0.001, differences between recognition index in the training and test session in the NOR. C, control group, C received saline; M, d-gal model group, M received 150 mg/kg d-gal; V, 100 mg/kg VE group, it received 100 mg/kg VE + 150 mg/kg d-gal; P-3, 3 mg/kg PAE group, it received 3 mg/kg PAE + 150 mg/kg d-gal; P-10, 10 mg/kg PAE group, it received 10 mg/kg PAE + 150 mg/kg d-gal; P-30, 30 mg/kg PAE group, it received 30 mg/kg PAE + 150 mg/kg d-gal.

The impact of treating with PAE on d-gal-induced learning and memory impairment was investigated in the novel object recognition (NOR). In the NOR test, a notable increase was observed in the object recognition index in the control group when compared with the test session with the training session (*p* < 0.01). However, the object recognition index was similar between the training and test in the d-gal-treated group, whereas pretreatment with vitamin E (VE) (100 mg/kg) and PAE (3, 10 and 30 mg/kg, oral) ameliorated the amnesic effect of d-gal (Figure 3A) (*p* < 0.01, *p* < 0.05, *p* < 0.01, *p* < 0.001). As observed, long-term treatment with d-gal could induce a recognition deficit in the NOR test, and this condition could be improved by pre-treatment with VE or PAE. Thus, PAE could improve the reference memory impairment induced by d-gal.

### 2.3. PAE Ameliorated the d-gal-Induced Working Memory Decline

**Figure 4:**
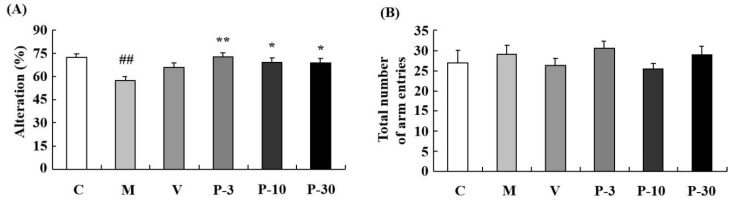
PAE effects on d-gal-induced memory impairment in the Y-maze test. The mice were placed at the end of one arm and allowed to explore in the Y-maze freely. The number of arm entries and sequence of arm visits was recorded manually for each mouse for 8 min. (**A**) The alternations and (**B**) total number of arm entries in the Y-maze. Results are presented as mean ± SEM. *n* = 9, ## *p* < 0.01 vs. vehicle control group and * *p* < 0.05, ** *p* < 0.01 vs. D-gal-treated group in the Y-maze. C, control group, C received saline; M, d-gal model group, M received 150 mg/kg d-gal; V, 100 mg/kg VE group, it received 100 mg/kg VE + 150 mg/kg d-gal; P-3, 3 mg/kg PAE group, it received 3 mg/kg PAE + 150 mg/kg d-gal; P-10, 10 mg/kg PAE group, it received 10 mg/kg PAE + 150 mg/kg d-gal; P-30, 30 mg/kg PAE group, it received 30 mg/kg PAE + 150 mg/kg d-gal.

The Y-maze test determined the influence of PAE on working memory. Relative to the control group, spontaneous alternation significantly decreased in the d-gal-treated group (Figure 4A) (*p* < 0.01). In addition, treatment with PAE (3, 10 and 30 mg/kg) obviously reversed the spontaneous alternation decline induced by the d-gal (Figure 4A) (*p* < 0.01, *p* < 0.05, *p* < 0.05). The results suggested that PAE could improve working memory and short-term memory. No significant differences were observed in total time spent on the object in the groups (Figure 3B) and arm entries amongst all the groups (Figure 4B). This result indicated that PAE did not influence the locomotor activity of the d-gal-induced ageing mice.

### 2.4. Effect of PAE on the Weight-Loaded Forced Swimming Test, the Body Weight and Organ Index

**Figure 5:**
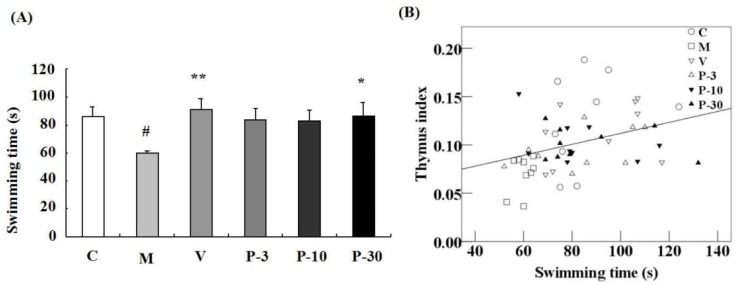
PAE effects on d-gal-induced fatigue in the weight-loaded forced swimming test (WLFST) and Pearson’s correlation between the swimming time versus thymus coefficients. The mice were individually placed into the plastic pool which containing freshwater. The mice were forced to swim in the water until exhaustion. The time that the mice were unable to keep their nose out of the water for 5 s was recorded as the exhausted swimming time. (**A**) The swimming times in the WLFST were recorded. Results are presented as mean ± SEM. *n* = 9, ## *p* < 0.01 vs. vehicle control group and * *p* < 0.05, ** *p* < 0.01 vs. d-gal-treated group in the WLFST. (**B**) Pearson’s correlation between the swimming time versus thymus coefficients was determined. C (hollow circle), M (hollow diamond), V (inverted hollow triangle), P-3 (hollow triangle), P-10 (inverted filled triangle), P-30 (filled triangle), *n* = 54.

We utilized a weight-loaded forced swimming test (WLFST) to observe the anti-fatigue effect of PAE. d-gal treatment triggered a significant decline of the swimming time compared with the control group in the WLFST (*p* < 0.05) (Figure 5A). In addition, treatment with VE or 30 mg/kg of PAE significantly reversed the swimming time decline caused by the d-gal (Figure 5A) (*p* < 0.01, *p* < 0.05). At the end of the experiment (Table 2), the thymus index of the d-gal-treated model group was also notably lower than that of the vehicle group (*p* < 0.01). Conversely, the parameter in VE or PAE at doses of 10 and 30 mg/kg plus d-gal treatment group was effectively ameliorated (*p* < 0.01, *p* < 0.05, *p* < 0.05). However, no evident difference was observed in the spleen index and body weight between the PAE treatment group and the d-gal model group (Table 2). The linear regression showed a significant positive correlation between the swimming time versus the thymus coefficient (*n* = 54, *r* = 0.344, *p* < 0.05; Figure 5B).

### 2.5. PAE Ameliorated Histopathological Alterations of the Liver, Spleen, and Brain

**Figure 6:**
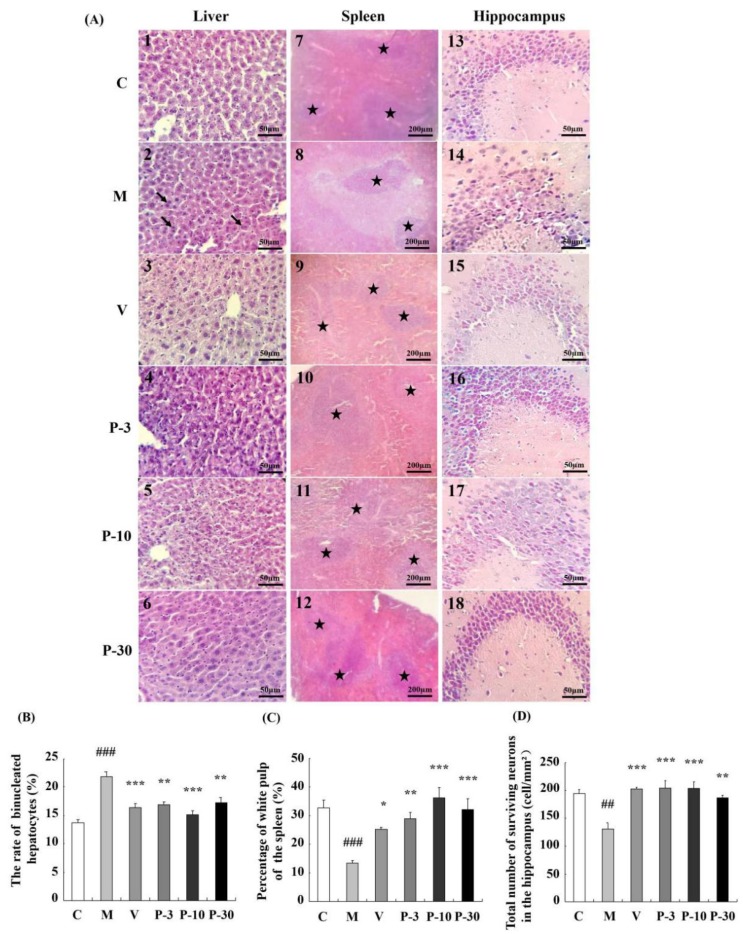
PAE attenuated histopathological alterations of the liver, spleen, and brain. (**A**) The histopathological changes of the liver, spleen, and brain (HE), the magnification of the liver and brain is 400, and spleen is 100; (**B**) the rate of binucleated hepatocytes; (**C**) spleen white pulp proportion; (**D**) surviving neurons of CA3. The black arrows show binucleated hepatocytes, ★ represents the white pulp area. The data are expressed as mean ± SEM, *n* = 6. ## *p* < 0.01 and ### *p* < 0.001 indicate significant difference compared with the control group; * *p* < 0.05, ** *p* < 0.01, and *** *p* < 0.001 indicate significant difference compared with the d-gal-treated control group. C, control group, C received saline; M, d-gal model group, M received 150 mg/kg d-gal; V, 100 mg/kg VE group, it received 100 mg/kg VE + 150 mg/kg d-gal; P-3, 3 mg/kg PAE group, it received 3 mg/kg PAE + 150 mg/kg d-gal; P-10, 10 mg/kg PAE group, it received 10 mg/kg PAE + 150 mg/kg d-gal; P-30, 30 mg/kg PAE group, it received 30 mg/kg PAE + 150 mg/kg d-gal.

Changes in the histopathology features of the liver, spleen, and brain sections as observed by haematoxylin and eosin (HE) staining are shown in Figure 6. Compared with the control group, the binuclear rate of the liver significantly increased in the d-gal group (Figure 6A2, *p* < 0.001), and treatment with PAE ameliorated such changes (Figure 6A4–6, *p* < 0.01, *p* < 0.001, *p* < 0.01). The percentage of the white-pulp area of the spleen significantly decreased in the d-gal-treated group (Figure 6A8, *p* < 0.001), and VE or PAE increased the percentage of the white-pulp area in relation to the d-gal-treated group (Figure 6A9–12, *p* < 0.05, *p* < 0.01, *p* < 0.001, *p* < 0.001). The histopathology alterations of the CA3 region of the hippocampus are shown in Figure 6A13–18. d-gal obviously decreased the number of surviving neurons in the CA3 region when compared with the control group (*p* < 0.01). VE or PAE also produced an obvious promotion on such change (*p* < 0.001, *p* < 0.001, *p* < 0.001, *p* < 0.01).

### 2.6. Effect of PAE on the Senescence-Associated β-galactosidase Activity of the Liver, Spleen, and Brain

**Figure 7:**
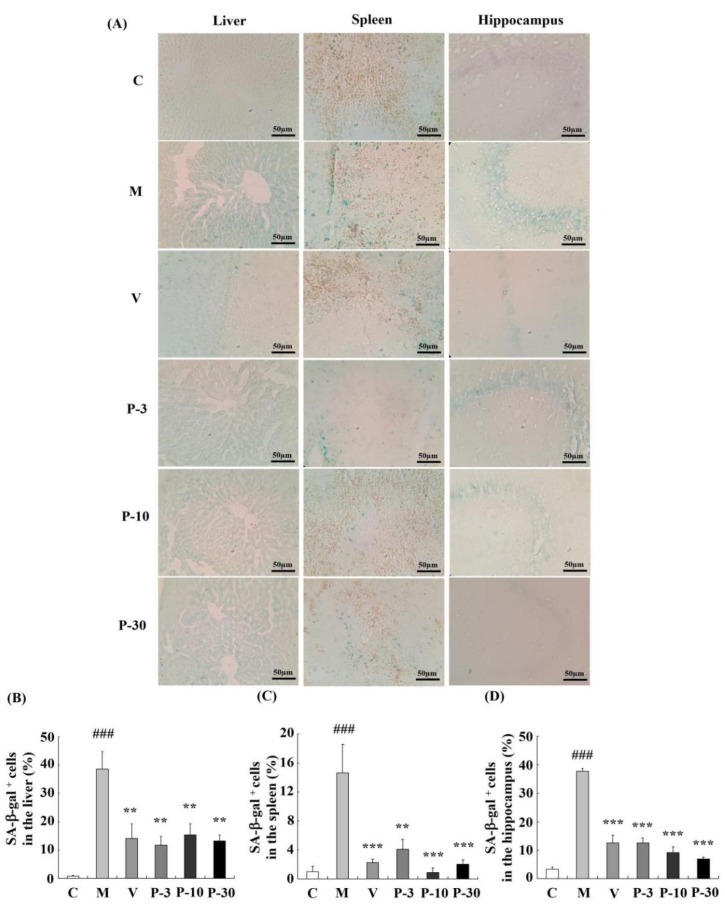
PAE effects on the activity of SA-β-gal in the liver, spleen, and brain. (**A**) The representative image of SA-β-gal staining in each group was captured (magnification 400×). (**B**–**D**) Quantification of the percentage of SA-β-gal-positive cell numbers in the liver, spleen, and hippocampus. Blue staining stands for SA-β-gal-positive cells. The data are expressed as mean ± SEM, *n* = 6. ### *p* < 0.001 indicate significant difference compared with the control group; ** *p* < 0.01, and *** *p* < 0.001 indicate significant difference compared with the d-gal-treated group. C, control group, C received saline; M, d-gal model group, M received 150 mg/kg d-gal; V, 100 mg/kg VE group, it received 100 mg/kg VE + 150 mg/kg d-gal; P-3, 3 mg/kg PAE group, it received 3 mg/kg PAE + 150 mg/kg d-gal; P-10, 10 mg/kg PAE group, it received 10 mg/kg PAE + 150 mg/kg d-gal; P-30, 30 mg/kg PAE group, it received 30 mg/kg PAE + 150 mg/kg d-gal.

To confirm the anti-ageing effect of PAE, we detected the most extensively recognized ageing biomarker and senescence-associated β-gal (SA-β-gal) activity in the liver, spleen, and brain. Related to the control group, the percentage of SA-β-gal positive cells in the liver, spleen, and brain increased greatly in the d-gal-treated group (represented by the blue staining in the images, Figure 7) (*p* < 0.001, *p* < 0.001, *p* < 0.001). These data confirmed the ageing changes of the liver, spleen, and brain. In addition, supplementation with VE or PAE showed effective inhibition of the percentage of SA-β-gal positive cells in the liver (*p* < 0.01 for all groups), spleen (*p* < 0.001, *p* < 0.01, *p* < 0.001, *p* < 0.001), and brain (*p* < 0.001 for all groups). These results suggested that the PAE effectively attenuates d-gal-induced ageing in mice.

### 2.7. Anti-Oxidative Stress Effect of PAE on the Liver, Plasma, and Brain of d-gal-Induced Ageing Mice

**Figure 8:**
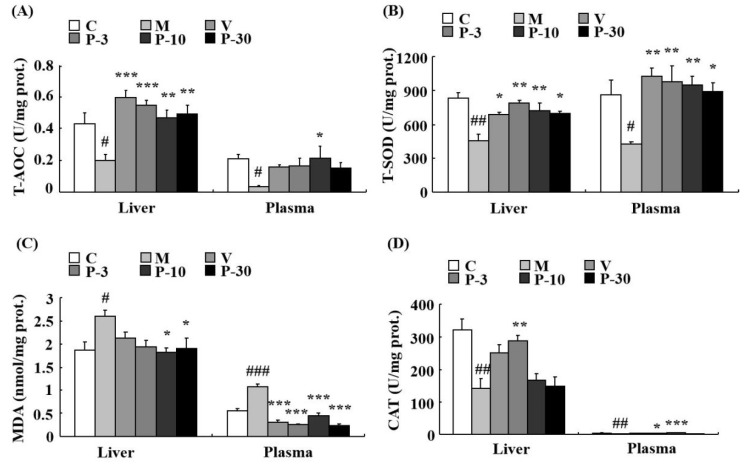
PAE decreased the oxidative stress in the liver and plasma. (**A**) Changes in total antioxidant capacity (T-AOC), (**B**) total superoxide dismutase (T-SOD) activity, (**C**) malondialdehyde (MDA) level, and (**D**) catalase (CAT) activity in the liver and plasma. Each value represents the mean ± SEM of 6 mice. # *p* < 0.05, ## *p* < 0.01 and ### *p* < 0.001 indicate significant difference compared with the control group; * *p* < 0.05, ** *p* < 0.01, and *** *p* < 0.001 indicate significant difference compared with the d-gal-treated group. C, control group, C received saline; M, d-gal model group, M received 150 mg/kg d-gal; V, 100 mg/kg VE group, it received 100 mg/kg VE + 150 mg/kg d-gal; P-3, 3 mg/kg PAE group, it received 3 mg/kg PAE + 150 mg/kg d-gal; P-10, 10 mg/kg PAE group, it received 10 mg/kg PAE + 150 mg/kg d-gal; P-30, 30 mg/kg PAE group, it received 30 mg/kg PAE + 150 mg/kg d-gal.

**Figure 9:**
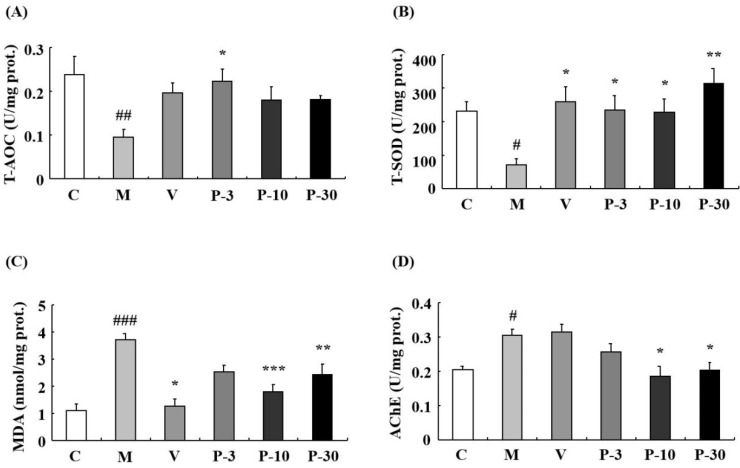
PAE effects on T-AOC, T-SOD, MDA, and acetylcholinesterase (AChE) activities in the brain. (**A**) Changes in T-AOC, (**B**) T-SOD activity, (**C**) MDA level, and (**D**) AChE activity in the brain. Each value represents the mean ± SEM of six mice. # *p* < 0.05, ## *p* < 0.01 and ### *p* < 0.001 indicate significant difference compared with the control group; * *p* < 0.05, ** *p* < 0.01, and *** *p* < 0.001 indicate significant difference compared with the d-gal-treated group. C, control group, C received saline; M, d-gal model group, M received 150 mg/kg d-gal; V, 100 mg/kg VE group, it received 100 mg/kg VE + 150 mg/kg d-gal; P-3, 3 mg/kg PAE group, it received 3 mg/kg PAE + 150 mg/kg d-gal; P-10, 10 mg/kg PAE group, it received 10 mg/kg PAE + 150 mg/kg d-gal; P-30, 30 mg/kg PAE group, it received 30 mg/kg PAE + 150 mg/kg d-gal.

We also investigated the anti-oxidative stress effect of PAE of d-gal-induced ageing mice. Total antioxidant capacity (T-AOC) was detected firstly in the liver, plasma, and brain. The data showed that compared with the control group, the d-gal obviously inhibited the T-AOC activity in the liver, plasma, and brain (*p* < 0.05, *p* < 0.05, *p* < 0.01). In the d-gal-treated group, the T-AOC activity was increased in the liver during treatment with VE at a dose of 100 mg/kg, PAE at doses of 3, 10, and 30 mg/kg (*p* < 0.001, *p* < 0.001, *p* < 0.01, *p* < 0.01), in the plasma when treated with PAE at a dose of 10 mg/kg (*p* < 0.05) and in the brain when treated with PAE at a dose of 3 mg/kg (*p* < 0.05) (Figure 8A and Figure 9A).

The results showed that related to the control group, the activity of total superoxide dismutase (T-SOD) was inhibited by d-gal in the liver (*p* < 0.01), plasma (*p* < 0.05), and brain (*p* < 0.05). In addition, related to the model mice, T-SOD was increased by pre-treatment with VE, PAE in the liver (*p* < 0.05, *p* < 0.01, *p* < 0.01, *p* < 0.05), plasma (*p* < 0.01, *p* < 0.01, *p* < 0.01, *p* < 0.05), and brain (*p* < 0.05, *p* < 0.05, *p* < 0.05, *p* < 0.01) at different concentrations (Figure 8B and Figure 9B). Meanwhile, d-gal notably increased the malondialdehyde (MDA) level in the liver (*p* < 0.05), plasma (*p* < 0.001), and brain (*p* < 0.001), whereas the MDA increase induced by d-gal was inhibited in the liver when pretreated with PAE at doses of 10 and 30 mg/kg (*p* < 0.05, *p* < 0.05), in the plasma when treated with VE or PAE at different concentrations (*p* < 0.001, *p* < 0.001, *p* < 0.001, *p* < 0.001) and in the brain after treating with VE or PAE at doses of 10 and 30 mg/kg (*p* < 0.05, *p* < 0.001, *p* < 0.01) (Figure 8C and Figure 9C).

The catalase (CAT) activity reduced in the d-gal-group in the liver (*p* < 0.01) and plasma (*p* < 0.01) but was significantly increased in the liver of the 3 mg/kg PAE group (*p* < 0.01) and in the plasma of the PAE at doses of 3 and 10 mg/kg (*p* < 0.05, *p* < 0.001) when compared with the d-gal group (Figure 8D). Then, we investigated the acetylcholinesterase (AChE) activity change of the brain. Compared with the control group, the d-gal notably increased the AChE activity of the brain (*p* < 0.05), and treatment with PAE at doses of 10 and 30 mg/kg inhibited the increase in AChE activity induced by d-gal (*p* < 0.05, *p* < 0.05) (Figure 9D).

## 3. Discussion

d-Gal can cause systematic ageing-related changes, such as hepatic and brain injury, a decline in immune function [26,27,28,29], learning and memory impairment, and fatigue in behavior studies [26,27]. Parallel histopathological features of the liver, spleen, and hippocampus were observed similarly in the d-gal-induced ageing and naturally aging groups [30]. The rate of binucleated hepatocytes increased with ageing [31]. d-Gal apparently increased the binuclear rate of the liver [32] but decreased the white-pulp proportion of spleen [33] and the surviving neurons of the hippocampus [34] in the previous studies. d-Gal-induced mimetic ageing is associated with an increase in oxidative stress [27,28]. Oxidative stress promoted cellular senescence [35]. A large number of studies have used the d-gal-induced ageing model for screening of anti-ageing compounds [26,27,28,29,32,33,34]. In our study, the d-gal-induced ageing model was well-established because it showed signs similar to the reported researches above. PAE could delay senescence on d-gal-induced ageing mice.

The NOR and Y-maze behavior tests were used to investigate the learning and memory improvement of PAE. The results showed that PAE could ameliorate the reference memory in NOR and working memory in the Y-maze. The hippocampus plays an important role in learning and memory, and the HE and SA-β-gal staining implied that PAE could maintain the survival cells and decrease the ageing cells of the hippocampus. The reduction of senescent cells could prevent tau-dependent pathology and improve the learning and memory decline, demonstrating that targeting senescent cells could be a therapeutic avenue for treating these pathologies, like AD [12]. Moreover, PAE could decrease the oxidative stress and the AChE activity of the brain. Changes in AChE activity are causally associated with learning and memory decline [36]. In addition, the brain is sensitive to oxidative stress, and oxidative stress could decrease neurogenesis and increase the death of neurons [37]. These data were consistent with the behavioral test results.

With ageing, fatigue prevalence increases for men and women and is related to adverse health outcomes, like morbidity and disability [3]. The WLFST is usually used to evaluate the anti-fatigue effect [38,39]. Thus, we utilized WLFST to observe the anti-fatigue effect of PAE. PAE enhanced the power of the ageing mice in the WLFST. This condition may be due to the improvement of the immune function of PAE. The previous study showed that fatigue is related to immune disorder [40]. d-Gal treatment reduced the thymus coefficient, and the white-pulp proportion of the spleen also increased the ageing cells in the spleen. However, PAE improved the situation, and Pearson’s correlation analysis showed a significant positive correlation between the swimming time and the thymus index. The results indicated that PAE produces an anti-fatigue effect through amelioration of the immune function.

The accumulation of senescent cells in various tissues is a typical feature of age and ageing-related disease in the recent report [13]. The cellular senescence has a causal relationship with age-related phenotypes, and reduction of senescent cells could delay tissue dysfunction and extend the healthspan of the mice [8]. The accumulation of senescent cells induced liver dysfunction, such as hepatic fat accumulation and liver steatosis, and the clearance of senescent cells of liver decreased hepatic steatosis globally [9]. In our study, overload of d-gal could increase senescent cells in the liver, brain, and spleen. Furthermore, PAE can protect against the senescence of the liver, spleen, and brain. These results suggest that PAE ameliorated the physical signs via inhibition of senescent cells.

Oxidative stress is one of the major factors of cellular senescence [41]. In the antioxidant enzyme defense system, VE, superoxide dismutase, and CAT are the primary members [42]. Firstly, superoxide dismutase transforms superoxide radical anions (O_2_^−^) into H_2_O_2_, and then CAT transforms the toxic H_2_O_2_ into H_2_O [43]. T-AOC implies the capacity of the non-enzymatic antioxidant defense system [44]. In our studies, the T-AOC and T-SOD obviously increased in the liver, plasma, and brain, and CAT increased significantly in the plasma and liver in the PAE-treated group compared with the d-gal treated group. MDA is considered an essential biomarker of oxidative stress in vivo [45]. The effect of PAE on d-gal-induced MDA level was tested, and results showed that PAE treatment notably decreased the MDA levels in the plasma, liver, and brain. These data indicated the antioxidant’s involvement in the anti-ageing effect of PAE.

The previous study implied that, once senescent cells generated, it would remain in the tissue over the lifespan. Thus, all sorts of oxidative stress through the lifespan potentially results in the accumulation of ageing cells [41]. Thus, the only reduction of oxidative stress is not enough. The effect on inhibition of senescent cells should be observed when evaluating the anti-ageing effect. In our studies, PAE could inhibit both oxidative stress and cellular senescence caused by d-gal.

The PAE contains an abundance of flavonoids, phenols, and saponins according to the analysis of bioactive components. In the previous studies, the saponins extracted from natural products could improve cognitive deficits in SAMP8 mice [46], protect splenocytes and thymocytes in an ageing rat model induced by d-gal [33], and attenuate d-gal-induced ageing in rats by activating FOXO3a and Nrf2 pathways [43]. Luteolin reduced inflammatory in the brain of senescent mice [47] and showed neuroprotective activity of various brain disorders [48], and rutin could also protect ageing-related metabolic dysfunction [49]. Several natural compounds existing with anti-aging effects were discovered in the past two decades, and such active compounds can be divided to saponins, flavonoids and phenols [50]. Thus, these bioactive components of PAE may contribute to its anti-ageing effect.

## 4. Materials and Methods

### 4.1. Sample Preparation

The calyx of *P.* *alkekengi* was purchased from a wholesale oriental herbal store in Jinzhong, Shanxi Province, China. The dried calyx (500 g) were smashed and macerated with 95% ethanol (10 L) for one week. The extract solution was subsequently evaporated in vacuo and concentrated to obtain the ethanol extract. The extract was partitioned between water and ethyl acetate (1:2), and the ethyl acetate layer was concentrated to obtain a 29.7 g dried sample.

### 4.2. Measurement of Total Flavonoid Content

The TFC of PAE was measured by the NaNO_2_-Al(NO_3_)_3_-NaOH colorimetric method by using rutin as the standard substance [51]. In a typical procedure, the sample or rutin was dissolved in 70% ethanol. The sample (500 μL) or rutin (0, 100, 200, 300, 400 and 500 μL) was mixed with 50 μL NaNO_2_ solution (5%) and reacted for 6 min at room temperature. Then, 50 μL of Al(NO_3_)_3_·9H_2_O solution (10%) was added and kept for 6 min. Finally, 500 μL NaOH solution (4%) and the 70% ethanol were added to obtain a final volume of 1.25 mL. After 15 min, the optical absorbance was measured at 510 nm. The TFC of PAE was calculated from the calibration curve.

### 4.3. Determination of Total Phenolic Content

The TPC was measured by the Folin–Ciocalteu method by using gallic acid (GA) as the standard substance [52]. In a typical procedure, the sample (50 μL) or GA (0, 10, 20, 30, 40, and 50 μL) was reacted with 500 μL Folin–Ciocalteu reagent (diluted tenfold) for 5 min. Then, 500 μL Na_2_CO_3_ solution (10%) and 70% ethanol solution were added to obtain a final volume of 1.05 mL. After reacting for 30 min in the dark at room temperature, the optical absorbance was measured at 765 nm, and the TPC of PAE was calculated from the calibration curve and expressed as milligram GAEs per gram PAE (mg GAEs/g).

### 4.4. Determination of Total Saponin Content

The TSC was determined by the vanillin–glacial acetic acid–sulphuric acid colorimetric method [53], and oleanolic acid (OA) was used as standard. The sample or OA was dissolved in absolute methanol. Then, the sample (100 μL) or OA (0, 20, 40, 60, 80 and 100 μL) was dried and mixed with 250 μL vanillin–glacial acetic acid solution (10%) and 2.5 mL sulphuric acid solution (60%). The mixture was reacted at 65 °C for 30 min and then cooled in ice water for 5 min. Subsequently, the optical absorbance was measured at 540 nm, and the TSC of PAE was calculated from the calibration curve and expressed as milligram OAEs per gram PAE (mg OAEs/g).

### 4.5. Quantification of Rutin and Luteolin by HPLC

Rutin and luteolin were dissolved in methanol to obtain a calibration solution (0.2 mg/mL), and the sample solution of PAE was prepared by dissolving 10 mg PAE in 1 mL methanol. The contents of rutin and luteolin in PAE were detected using Dionex Ultimate 3000 (Thermo Fisher Scientific, Waltham, MA, USA) under the following conditions: Agela C18 column (4.6 × 250 mm, 5 μm), detection wavelength: 250 nm, *t* = 0 min MeOH/H_2_O/formic acid (40:60:0.4) and *t* = 50 min MeOH/H_2_O/formic acid (90:10:0.4), flow rate: 1 mL/min, the injection volume: 10 µL and the column temperature: 30 °C. The result was analyzed by the Chromeleon Dionex data processing system (Thermo Fisher Scientific, Waltham, MA, USA).

### 4.6. Chemicals and Kits 

VE, OA (> 98%), GA (> 98%), luteolin, vanillin, and Al(NO_3_)_3_·9H_2_O were purchased from Aladdin Reagent Company (Shanghai, China). d-Gal and Folin–Ciocalteu were purchased from Beijing Solarbio Science and Technology Company (Beijing, China). Rutin (> 98%) was purchased from Guizhou Dida Technology Co., Ltd. (Guizhou, China). All other materials were the highest grade available.

Kits for T-AOC, SOD, MDA, CAT and AChE were purchased from the Nanjing Jiancheng Institute of Biotechnology (Nanjing, China). A protein assay kit was bought from Bio-Rad Laboratories (Hercules, CA, USA), and the HE kit was purchased from Beijing Solarbio Science and Technology Company (Beijing, China). A senescence β-gal staining kit was obtained from the Beyotime Institute of Biotechnology (Haimen, China).

### 4.7. Animals and Drug Administration

The 7-week-old male Institute of Cancer Research mice (weighing 28–30 g) purchased from Shanxi Medical University (Shanxi, China) were fed to the adaption of the environment for a week. The mice were fed to access food and water freely and housed in the constant temperature (23 ± 1 °C) and relative humidity (55% ± 5%) under 12 h:12 h light/dark cycle. The investigation conformed to the Guide for the Care and Use of Laboratory Animals published by the United States National Institute of Health (Publication No. 85–23, revised, 1996, https://www.nap.edu/catalog/5140/guide-for-the-care-and-use-of-laboratory-animals). All the animals were treated according to the Guide for the Care and Use of Laboratory Animals of Shanxi Agricultural University. The protocol and operation were approved by the Institutional Animal Care and Use Committee of Shanxi Agricultural University (number: SXAU-EAW-2019-M002004, 12 March 2019).

After acclimating for one week, the mice were weighed again as the initial weight and randomly divided into six groups as follows (nine mice in one group): the control group, model group (150 mg/kg d-gal), positive group (100 mg/kg VE), PAE low-dose group (3 mg/kg PAE), PAE middle-dose group (10 mg/kg PAE), and PAE high-dose group (30 mg/kg PAE). The control group was injected once daily with saline, and the other groups were treated with once-daily d-gal (150 mg/kg) by subcutaneous injection for the first six weeks. From the fifth week, the VE or PAE groups were subjected to VE (100 mg/kg) or PAE (at doses of 3, 10, and 30 mg/kg, oral) once daily for five weeks, and the control group and model group were administered to solvent (2% ethanol in oil, oral) in the same way instead. In the ninth week, the behavior tests were investigated after PAE administration. 

### 4.8. Novel Object Recognition Task 

The recognition learning and memory abilities were detected by a NOR task according to a method described previously [54]. In a typical procedure, the task contains an adaption and test period. In the two-day adaption period, mice facing the wall had been placed into the apparatus for 5 min without objects. On the third-day test period, the mice were detected by comparing two sessions: the training and test sessions. The mice were placed into the apparatus containing two identical objects and allowed to explore for 5 min in the training session. After 60 min, a novel object replaced one of the two familiar objects, and then the test session was performed. Discrimination ratio is defined as TN/(TN + TF) ratio [TF = exploring time on the familiar object; TN = exploring time on the novel object].

### 4.9. Spontaneous Alternation Behavior Y-maze Test 

The Y-maze test was performed as described previously [54]. In a typical procedure, the mice were placed at the end of one arm and allowed to explore in the Y-maze freely. The number of arm entries and sequence of arm visits was recorded manually for each mouse at 8 min. The mouse entering into three different arms in succession was defined as an actual alteration, i.e., ABC, BCA, or CAB but not BBC. In addition, the percentage of alteration was calculated as the number of actual alterations/(total arm entries-2) × 100.

### 4.10. Weight-Loaded Forced Swimming Test 

The WLFST is used to evaluate the anti-fatigue effect of PAE, and the experiment was performed as described previously [55]. The mice were individually placed into the plastic pool (30 cm × 35 cm × 60 cm) containing freshwater (23 ± 1 °C), approximately 50 cm deep. The mice were loaded with aluminium, which weighs 5% of their body weight, and was attached to the tail. Before the experiment, the mice were dripping wet with water. The time that the mice were unable to keep their noses out of the water for 5 s was recorded as the exhausted swimming time.

### 4.11. Bodyweight Measurement and Organ Coefficient Analysis

After the adaption, the mice were weighted as initial weight. After the weight-loaded forced swimming test, the mice were injected with PAE or solvent for the last administration. Then, the mice were weighted as the final weight, anesthetized, and killed. The thymus and spleens were removed and weighed for organ coefficient calculation. Blood samples were obtained for biochemical assays, and half of the whole brain and part of the liver and spleen were used for histopathological evaluation. The remaining part was used for biochemical assays. The organ coefficient was calculated as follows: coefficient (g/g) = organ weight (g)/body weight (g) × 100.

### 4.12. Morphological and Histological Analyses

The liver, brain and spleen tissues were fixed in 4% paraformaldehyde and embedded in paraffine. The tissues were cut into 5 μm slices. The slices were stained with HE, and all the sections were deparaffinized with 100% xylene and then rehydrated with gradient alcohol (100% ethanol, 90% aqueous ethanol, 80% aqueous ethanol, 70% aqueous ethanol and distilled water). Then, the sections were stained with the haematoxylin for 4, 3, and 5 min, and the nucleus was counterstained with eosin for 40, 90, and 60 s for the liver, brain, and spleen, respectively, and dehydrated. Then, the pathological changes were observed with an optical microscope (Olympus, Tokyo, Japan). For each individual, three random sections of the spleen were selected. The percentage of the white-pulp area of the spleen was then measured using Image J (National Institutes of Health, Bethesda, MD, USA) through the following formula: percentage of white-pulp area = white-pulp area/total area × 100.

### 4.13. SA-β-gal Staining

The SA-β-gal positive cells were stained using a senescence β-gal staining kit. In a typical procedure, 10 μm slices were washed with phosphate-buffered saline for 5 min (thrice) and then incubated in SA-β-gal staining solution at 37 °C without CO_2_ according to the protocol for 48 h. Then, the slices were washed with phosphate-buffered saline for 5 min (thrice) and coverslipped. The SA-β-gal positive cells were observed with a microscope (Olympus, Tokyo, Japan). The percentage of SA-β-gal positive cells was calculated as the number of SA-β-gal positive cells/total number of cells.

### 4.14. Biochemical Analysis

Before detection, the tissues were weighed, and then ice-cold saline (nine times the tissue weight) was added into the tissues. The tissues were subsequently rapidly homogenized with a homogenizer, and the homogenates were centrifuged at 4000 g at 4 °C for 15 min. Subsequently, the supernatant was collected for biochemical analysis. Protein concentrations were determined with a protein assay kit. The enzyme activities and MDA levels were measured according to the protocols of detection kits. The activities of T-AOC, T-SOD, CAT, and AChE were expressed in U/mg protein, and the MDA level was shown in nmol/mg protein.

### 4.15. Statistical Analyses

The results of the Y-maze, WLFST, organ index, enzyme activities, and MDA level are expressed as mean ± standard error of the mean. Statistical analysis was conducted by a one-way ANOVA. For the NOR, statistical evaluation was performed by a paired sample *t*-test. *p* < 0.05 was considered statistically significant.

## 5. Conclusions

PAE alleviated d-gal-induced ageing-related changes, such as learning and memory decline, fatigue, and immune dysfunction. Moreover, PAE protected from liver and brain disorders by inhibiting both oxidative stress and cellular senescence. Therefore, PAE could be a candidate for the prevention or treatment of ageing-relative diseases.

## Figures and Tables

**Table 1:** The bioactive components of PAE.

Bioactive Components	Content
TFC (mg REs/g PAE)	71.72 ± 2.99
TPC (mg GAEs/g PAE)	40.19 ± 0.47
TSC (mg OAEs / g PAE)	128.13 ± 1.04
Rutin (mg/ g PAE)	1.67 ± 0.07
Luteolin (mg/g PAE)	1.62 ± 0.01

TPC, Total phenolic content; TFC, Total flavonoid content; TSC, Total saponins content.

**Table 2:** Effect of PAE on organ index, body weight.

	Weight (g)		Organ Index (*g*/*g* %)	
Group	Initial	Final	Thymus	Spleen
Control	34.8 ± 2.1	40.3 ± 2.0	0.126 ± 0.016	0.383 ± 0.018
d-gal	33.3 ± 1.3	38.7 ± 0.7	0.070 ± 0.006 ##	0.401 ± 0.019
VE-100	35.2 ± 2.0	38.6 ± 1.4	0.112 ± 0.011 **	0.422 ± 0.057
PAE-3	34.8 ± 1.6	40.0 ± 0.8	0.095 ± 0.007	0.407 ± 0.030
PAE-10	34.7 ± 1.9	40.2 ± 1.4	0.103 ± 0.007 *	0.410 ± 0.014
PAE-30	35.4 ± 1.7	38.1 ± 1.0	0.102 ± 0.006 *	0.417 ± 0.018

The data are expressed as mean ± SEM. *n* = 9, ## *p* < 0.01 vs. vehicle control group and * *p* < 0.05, ** *p* < 0.01 vs the d-gal-treated control group.

## Abbreviations

AChE	Acetylcholinesterase
AD	Alzheimer’s disease
CAT	Catalase
d-gal	d-galactose
GA	Gallic acid
GAEs	Gallic acid equivalents
HE	Haematoxylin and eosin
HPLC	High-performance liquid chromatography
MDA	Malondialdehyde
NOR	Novel object recognition
OA	Oleanolic acid
OAEs	Oleanolic acid equivalents
PAE	Ethyl acetate fraction from *Physalis Alkekengi*
REs	Rutin equivalents
ROS	Reactive oxygen species
SA-β-gal	Senescence-associated β-galactosidase
T-AOC	Total anti-oxidant capacity
T-SOD	Total superoxide dismutase
TFC	Total phenolic content
TPC	Total phenolic content
TSC	Total saponin content
VE	Vitamin E
WLFST	Weight-loaded forced swimming test
